# An evaluation of the Hitachi 705 analyser

**DOI:** 10.1155/S1463924684000080

**Published:** 1984

**Authors:** C. Ferré, M. J. Castiñeiras, M. J. Alsina, R. Galimany, J. Riera

**Affiliations:** Departamento de Análisis Clínicos Residencia Sanitaria S.S. ‘Príncipes de España’ Hospitalet de Llobregat Barcelona Spain


Journal of Automatic Chemistry, Volume 6, Number 1 (January–March 1984), pages 39–43

### An evaluation of the Hitachi 705 analyser

**C. Ferré, M. J. Castiñeiras, M. J. Alsina, R. Galimany and J. Riera**

*Departamento de Análisis Clínicos, Residencia Sanitaria S.S. 'Príncipes de España', Hospitalet de Llobregat, Barcelona, Spain*

### Introduction

The Hitachi 705 analyser was evaluated for three months, in line with the recommendations of the 'Sociedad Española de Química Clínica' (SEQC) [1].

A 10-channel configuration was selected to suit the requirements of the emergency laboratory in which the instrument was installed, and to represent different types of chemical determinations. The analyses performed were alpha-amylase (AMYL), creatin kinase (CK), creatin kinase isoenzyme MB (CK-MB), alanine aminotransferase (ALT), aspartate aminotransferase (AST), glucose (GLU), urea (UREA), creatinine (CREA), calcium (CA), and protein (TP). (The electrolytes analyser assembly was not available at the time of evaluation.) The aspects evaluated included imprecision at three different levels; carry-over; linearity; and relative accuracy when compared with the laboratory's routine analysers: an SMAC autoanalyser (Technicon Instrument Corporation, Tarrytown, New York, USA), and an ABA-100 (Abbott Laboratories, South Pasadena, California, USA).

### Materials and methods

### *The instrument*

The Hitachi 705 is a compact, discrete, selective multianalyser for clinical chemistry laboratories. The instrument is capable of simultaneously performing up to 16 tests/sample, plus sodium, potassium and chloride measurements, which are always made together (with an optional accessory). The sample throughput is 180 tests/h, or 220 tests/h if electrolytes are included. The routine procedure can be interrupted by stat samples at any time, returning afterwards to the original sequence. Sodium, potassium and chloride determinations are performed by ion-selective permeable microelectrodes with dialytic cellulose membranes for $Na^+$, $K^+$, and $Cl^-$. The analytical method for biochemical determinations employs either endpoint or rate assays with a single reagent or two reagents. An endpoint assay has a maximum reaction time of 10 min, with the possibility of a sample blank correction. Kinetic assays may be performed freely.

Routine sample, stat samples, standard solutions, a blank solution and control sera are loaded on the sample disc. The serum and reagent pipettes are driven by a stepper motor and are supplied with a probe-washing device and a liquid-level sensor. The sample probe aspirates the serum (from 5 to 20 $\mu$l) and discharges it into each reaction cuvette on the reaction disc and/or into the $Na^+$, $K^+$, $Cl^-$ measuring vessel. (The sample volume required for the electrolytes is 100 $\mu$l.) The reagent pipetting unit picks up the required volume of reagent (from 100 to 500 $\mu$l) from its bottle in the refrigerated reagent store. The reagent is heated in the pipettes to the specified incubation temperature and is then discharged into the reaction cells containing the serum samples. The incubation water-bath has a temperature range of 25–37°C. The reaction disc, which holds 48 semidisposable reaction cuvettes, rotates so that each cuvette crosses the optical path of the spectrophotometer (11 fixed wavelengths between 340 and 700 nm); absorbance is measured directly at two wavelengths.

Measurements are taken up to 31 times at 20 s intervals over a period of 10 min after the addition of the first reagent. When each sample has been measured, its reaction cuvette is thoroughly washed. The washing unit includes a facility for measuring the absorbance of the reaction cuvettes once they have been refilled with distilled water. The value obtained is stored and used as a reference value for the next measurement.

The analyser is controlled by a computer with which the operator communicates by means of a keyboard, a visual-display unit and a magnetic tape. Up to 40 analytical methods can be programmed into the computer.

All analytical methods are open to variation and are stored on tape. The functions of the microprocessor are many, including selecting the requested test for each sample. Concentration results are printed out after compensation for the reaction cuvette absorbance, photometer drift and self blank, and arithmetic operations on values obtained in a rate assay, endpoint assay, single and double assay. It will automatically establish a calibration curve for any parameter. It monitors the average value, normal value range, standard deviation and coefficient of variation for three types of control sera. It is equipped with an alarm unit to warn the operator about substrate exhaustion, reagent shortage, errors, and malfunctions. In addition, it can be connected on line to an external computer system.

### *Analytical methods and reagents*

Alpha-amylase was assayed using a colorimetric test with p-nitrophenyl-alpha-D-maltoheptoside as substrate [2]. Creatin kinase, alanine aminotransferase and aspartate aminotransferase were measured by the optimized standard methods recommended by the Deutsche Gessellschaft für Klinische Chemie [3–6]. Creatin kinase isoenzyme MB was measured using an immuno-inhibition assay test [7], and creatinine by the Jaffé kinetic method without deproteinization [8]. The GOD-PAP method [9] was used for glucose. The urease-GLDH assay [10] was adapted to analyse urea. The colorimetric method with o-cresolphthalein complexone without deproteinization [11] was used to measure calcium. Protein assay was carried out by the Biuret method with sample blank [12]. Analytical details are shown in table 1. The working reagents, solutions and calibrators were supplied by Boehringer Mannheim GmbH. The control sera used were Validate (General Diagnostics), Sera Chem (Fisher Diagnostics), Precinorm-U, Precipath-U, and CK-MB control (Boehringer Mannheim GmbH).

### *Standardization*

Standardization was normally undertaken at the start of each day following the instructions in the instrument's manual. The reagent blanks were measured for all the parameters.

*Table 1. Details of methods used for measurements on the Hitachi 705. (Where $\lambda_1$ = peak wavelength and $\lambda_2$ = side band wavelength.)*

| | Sample volume (μl) | Total volume (μl) | Analytical method | Wavelength (nm) $\lambda_1$ | Wavelength (nm) $\lambda_2$ | Temperature (°C) |
|---|---|---|---|---|---|---|
| Alpha-amylase | 10 | 430 | Kinetic | 415 | 660 | 30 |
| Creatin kinase | 10 | 430 | Kinetic | 340 | 376 | 30 |
| Creatin kinase-MB | 15 | 405 | Kinetic | 340 | 376 | 30 |
| Aspartate aminotransferase | 20 | 440 | Kinetic | 340 | 376 | 30 |
| Alanine aminotransferase | 20 | 440 | Kinetic | 340 | 376 | 30 |
| Glucose | 5 | 505 | End point | 600 | 546 | 30 |
| Urea | 5 | 360 | Fixed time | 340 | 376 | 30 |
| Creatinine | 20 | 440 | Kinetic | 505 | 570 | 30 |
| Calcium | 10 | 410 | End point | 600 | 660 | 30 |
| Protein | 10 | 410 | End point | 340 | 376 | 30 |

Calibration curves were produced for glucose, creatinine, calcium, protein, urea, and alpha-amylase, using Precinorm-U as the calibrator (the Precinorm-U used as calibrator was different from that used as the control). A factor, obtained after several calibrator analyses, was applied to creatin kinase, creatin kinase isoenzyme MB, alanine aminotransferase, and aspartate aminotransferase determinations.

### *Imprecision*

The within-day imprecision was evaluated by analysing control sera at three different levels of concentration 30 times within a single batch (within one calibration).

The day-to-day imprecision was determined using frozen aliquots of control sera with three different levels of analyte which were analysed each day for 30 working days.

### *Linearity*

A serum with a high concentration of the analyte in question was diluted with saline or with another serum containing a relatively low level of that analyte; so mixtures with linearly-related concentrations were obtained. These were used for linearity studies.

### *Carry-over*

The features of the instrument required studies of the possibilities of contamination in the sample probe, reagent pipette, and reaction cuvettes.

### *Sample probe carry-over*

Control sera at three different levels of concentration (H = high, M = medium, L = low) were used for each analyte and processed according to the SEQC's recommendations (table 2). The Snedecor F-test ($\alpha$ 0·05) was used to assess contamination between samples of different concentrations for each analyte. The variance of all the results was compared for each analyte at each level with the variance of the results obtained from samples not susceptible to carry-over.

### *Reagent pipette carry-over*

To study reagent-to-reagent carry-over one analyte was alternated with the others, as shown in the sequence for creatin kinase:

AMYL/CK/CK/CK/CK-MB/CK/AST/CK/ALT/CK/GLU/CK/UREA/CK/CREA/CK/CA/CK/TP/CK.

The coefficient of variation for the measurement of creatin kinase under these circumstances was then calculated. Similar sequences were then prepared for each analyte, using the same control sera, and the corresponding coefficients of variation were calculated.

### *Reaction cuvette carry-over*

Cuvette contamination was assessed according to the protocol of Knedel [13]; this allows determination of whether one analyte is affected by another when the same reaction cuvette is used. After employing the sequence: AMYL/CK/CK-MB/AST/ALT/GLU/UREA/CREA/CA/TP, the same 10 cuvettes are used to assay only one of the analytes. The procedure is repeated for each analyte, thus allowing calculation of the corresponding coefficients of variation.

### *Relative accuracy*

Glucose, urea, creatinine, calcium, and protein in sera from 110 patients were assayed by the Hitachi 705, and the results obtained were compared with those from the SMAC analyser using the same sera. The analytical procedures used by the SMAC are described elsewhere [14].

*Table 2. Sample sequence for measuring sample/sample carry-over according to the SEQC. (H, M, and L = high, medium, and low concentration levels of analyte respectively.)*

| | | | |
|---|---|---|---|
| H | L* | H* | L* |
| H* | L* | L | H |
| H* | L* | L* | H* |
| H | H | H | H* |
| L | H* | H* | L |
| L* | H* | H* | L* |
| M | M | M | L* |
| M* | M* | M* | L* |
| M* | M* | M* | L* |
| L | H | L | H |
| H | L | H | H* |
| H* | L* | H* | H* |
| L | L* | L | M |
| L* | H | L* | M* |
| L* | H* | L* | M* |

* = uncontaminated test levels.

*Table 3. Within-day imprecision at three concentrations.*

| | *N* | Mean | CV % |
|---|---|---|---|
| Alpha-amylase (U/l) | 30 | 40·0 | 4·3 |
| | 30 | 264·3 | 1·0 |
| | 30 | 714·7 | 1·6 |
| Creatin kinase (U/l) | 30 | 33·3 | 2·9 |
| | 30 | 81·7 | 1·6 |
| | 30 | 257·0 | 0·6 |
| Creatin kinase-MB (U/L) | 30 | 13·8 | 8·8 |
| | 30 | 39·0 | 3·2 |
| | 30 | 111·7 | 1·4 |
| Aspartate aminotransferase (U/l) | 30 | 12·8 | 3·2 |
| | 30 | 36·5 | 1·4 |
| | 30 | 134·0 | 0·4 |
| Alanine aminotransferase (U/l) | 30 | 19·5 | 2·6 |
| | 30 | 52·4 | 0·9 |
| | 30 | 126·0 | 0·5 |
| Glucose (mmol/l) | 30 | 3·2 | 1·6 |
| | 30 | 4·9 | 1·6 |
| | 30 | 12·9 | 0·5 |
| Urea (mmol/l) | 30 | 3·7 | 0·8 |
| | 30 | 7·0 | 1·0 |
| | 30 | 14·0 | 0·6 |
| Creatinine (μmol/l) | 30 | 65·6 | 2·9 |
| | 30 | 92·9 | 2·3 |
| | 30 | 302·0 | 0·9 |
| Calcium (mmol/l) | 30 | 1·6 | 0·9 |
| | 30 | 2·4 | 0·7 |
| | 30 | 3·1 | 0·5 |
| Protein (g/l) | 30 | 39·9 | 0·9 |
| | 30 | 59·7 | 1·1 |
| | 30 | 80·4 | 0·9 |

*Table 4. Day-to-day imprecision at three concentrations.*

| | *N* | Mean | CV % |
|---|---|---|---|
| Alpha-amylase (U/l) | 30 | 54·1 | 4·2 |
| | 30 | 282·3 | 1·4 |
| | 30 | 1153·2 | 1·0 |
| Creatin kinase (U/l) | 30 | 51·0 | 5·2 |
| | 30 | 70·9 | 4·4 |
| | 30 | 340·5 | 3·8 |
| Creatin kinase-MB (U/l) | 30 | 14·3 | 8·9 |
| | 30 | 33·9 | 4·0 |
| | 30 | 111·1 | 3·3 |
| Aspartate aminotransferase (U/l) | 30 | 11·8 | 4·5 |
| | 30 | 33·9 | 4·0 |
| | 30 | 151·1 | 3·3 |
| Alanine aminotransferase (U/l) | 30 | 22·5 | 3·6 |
| | 30 | 41·6 | 3·0 |
| | 30 | 148·0 | 2·5 |
| Glucose (mmol/l) | 30 | 3·2 | 3·1 |
| | 30 | 4·6 | 2·3 |
| | 30 | 13·9 | 1·4 |
| Urea (mmol/l) | 30 | 3·8 | 2·9 |
| | 30 | 4·7 | 2·5 |
| | 30 | 16·5 | 2·2 |
| Creatinine (μmol/l) | 30 | 61·6 | 4·0 |
| | 30 | 80·4 | 2·3 |
| | 30 | 555·0 | 1·6 |
| Calcium (mmol/l) | 30 | 1·6 | 2·0 |
| | 30 | 2·4 | 1·4 |
| | 30 | 3·1 | 1·3 |
| Protein (g/l) | 30 | 48·8 | 2·0 |
| | 30 | 62·6 | 2·0 |
| | 30 | 78·8 | 1·8 |

The alpha-amylase comparison was done with the ABA-100 analyser using 50 patient sera; the analytical reaction was the same as the Hitachi 705's.

### Results and discussion

### *Imprecision*

Within-day imprecision at each level studied is shown in table 3. Day-to-day imprecision is given in table 4.

The results show a remarkably low coefficient of variation at low, medium, and high levels of concentration for the analytes. The disappointing imprecision obtained at the low level for creatin kinase isoenzyme MB may be due at the very low increment of absorbances produced by such low catalytic concentrations.

### *Linearity*

The linearity for each constituent was assessed by visual inspection of the plot. Linearity for glucose and urea was up to 30 mmol/l, alpha-amylase up to 745 U/l, creatin kinase and creatin kinase isoenzyme MB up to 1600 U/l, aspartate and alanine aminotransferase up to 1000 U/l, creatinine up to 1000 μmol/l, calcium up to 4 mmol/l and protein up to 100 g/l.

*Table 5. Sample probe carry-over for glucose.*

| Results (mmol/l)—permutation | | | |
|---|---|---|---|
| 13·0 H | 3·1 L* | 13·1 H* | 3·2 L* |
| 13·1 H* | 3·1 L* | 3·2 L | 13·0 H |
| 13·1 H* | 3·1 L* | 3·2 L* | 13·1 H* |
| 13·1 H* | 13·2 H | 13·2 H | 13·2 H* |
| 3·2 L | 13·2 H* | 13·1 H* | 3·3 L |
| 3·1 L* | 13·1 H* | 13·3 H* | 3·2 L* |
| 5·1 M | 5·1 M | 5·2 M | 3·1 L |
| 5·0 M* | 5·2 M* | 5·1 M* | 3·1 L* |
| 5·0 M* | 5·1 M* | 5·2 M* | 3·2 L* |
| 3·1 L | 13·2 H | 3·1 L | 13·2 H |
| 13·1 H | 3·2 L | 13·1 H | 13·1 H* |
| 13·1 H* | 3·2 L* | 13·2 H* | 13·1 H* |
| 3·1 L | 3·2 L* | 3·1 L | 5·1 M |
| 3·2 L* | 13·1 H | 3·3 L* | 5·2 M* |
| 3·1 L* | 13·0 H* | 3·1 L* | 5·2 M* |

* = uncontaminated values.

*Table 6. Sample probe carry-over for glucose. $SD_1$ = standard deviation for all the glucose values ($n_1$) including the effects of contamination. $SD_2$ = standard deviation of glucose values ($n_2$) without the effects of contamination.*

| Level | $n_1$ | $n_2$ | $\bar{x}$ | $SD_1$ | $SD_2$ | Snedecor's value |
|---|---|---|---|---|---|---|
| High | 24 | 15 | 13·1 | 0·073 | 0·070 | 1·10 |
| Medium | 12 | 8 | 5·12 | 0·075 | 0·088 | 1·37 |
| Low | 24 | 15 | 3·16 | 0·070 | 0·072 | 1·04 |

*Table 7. Coefficients of variation from reagent pipette and from reaction cuvette carry-over studies compared with the imprecision coefficients.*

| | Reagent pipette CV % | Reaction cuvette CV % | Within-day precision CV % |
|---|---|---|---|
| | | 2·3 | 4·3 |
| Alpha-amylase | 0·3 | 1·4 | 1·0 |
| | | 0·5 | 1·6 |
| | | 2·6 | 2·9 |
| Creatin kinase | 0·5 | 1·3 | 1·6 |
| | | 0·6 | 0·6 |
| | | 9·0 | 8·8 |
| Creatin kinase-MB | 1·5 | 3·6 | 3·2 |
| | | 1·6 | 1·4 |
| | | 3·0 | 3·2 |
| Aspartate aminotransferase | 0·8 | 1·6 | 1·4 |
| | | 0·4 | 0·4 |
| | | 2·4 | 2·6 |
| Alanine aminotransferase | 0·6 | 0·9 | 0·9 |
| | | 0·6 | 0·5 |
| | | 0·6 | 1·6 |
| Glucose | 0·4 | 0·9 | 1·6 |
| | | 0·6 | 0·5 |
| | | 1·2 | 0·8 |
| Urea | 0·6 | 0·6 | 1·0 |
| | | 0·4 | 0·6 |
| | | 1·8 | 2·9 |
| Creatinine | 1·0 | 1·3 | 2·3 |
| | | 0·5 | 0·9 |
| | | 1·2 | 0·9 |
| Calcium | 0·6 | 1·0 | 0·7 |
| | | 0·9 | 0·5 |
| | | 1·2 | 0·9 |
| Protein | 0·8 | 1·2 | 1·1 |
| | | 1·0 | 0·9 |

### Carry-over

### Sample probe carry-over

In no case was there any significant difference between the compared variances. For each analyte at each of the three levels the method was the same as the glucose case shown in tables 5 and 6.

### Reagent pipette carry-over

The coefficients of variation obtained in the reagent pipette carry-over were similar to those obtained in the imprecision study (table 7). The procedure followed for each analyte was as the creatin kinase example (table 8). No appreciable carry-over was detected.

*Table 8. Reagent pipette carry-over for creatin kinase.*

| | | | |
|---|---|---|---|
| AMYL | (U/l) | 684·7 | |
| CK | (U/l) | 258 | |
| CK | (U/l) | 260 | |
| CK | (U/l) | 258 | |
| CK-MB | (U/l) | 111 | |
| CK | (U/l) | 257 | |
| AST | (U/l) | 130 | $n_{CK}$ = 11 |
| CK | (U/l) | 257 | $\bar{x}$ = 258 |
| ALT | (U/l) | 127 | SD = 1·19 |
| CK | (U/l) | 258 | CV % = 0·46 |
| GLU | (mmol/l) | 12·2 | |
| CK | (U/l) | 260 | |
| UREA | (mmol/l) | 13·4 | |
| CK | (U/l) | 258 | |
| CREA | (μmol/l) | 298 | |
| CK | (U/l) | 257 | |
| CA | (mmol/l) | 3·0 | |
| CK | (U/l) | 258 | |
| TP | (g/l) | 80 | |
| CK | (U/l) | 260 | |

*Table 9. Reaction cuvette carry-over for alpha-amylase.*

| Cuvette | First run | | | Second run | |
|---|---|---|---|---|---|
| 1 | AMYL | (U/l) | 684·7 | AMYL | 264·3 |
| 2 | CK | (U/l) | 258 | AMYL | 264·3 |
| 3 | CK-MB | (U/l) | 110 | AMYL | 264·3 |
| 4 | AST | (U/l) | 130 | AMYL | 264·3 |
| 5 | ALT | (U/l) | 128 | AMYL | 264·3 |
| 6 | GLU | (mmol/l) | 12·0 | AMYL | 270·3 |
| 7 | UREA | (mmol/l) | 13·4 | AMYL | 270·3 |
| 8 | CREA | (μmol/l) | 297 | AMYL | 264·3 |
| 9 | CA | (mmol/l) | 2·9 | AMYL | 270·3 |
| 10 | TP | (g/l) | 79·6 | AMYL | 264·3 |

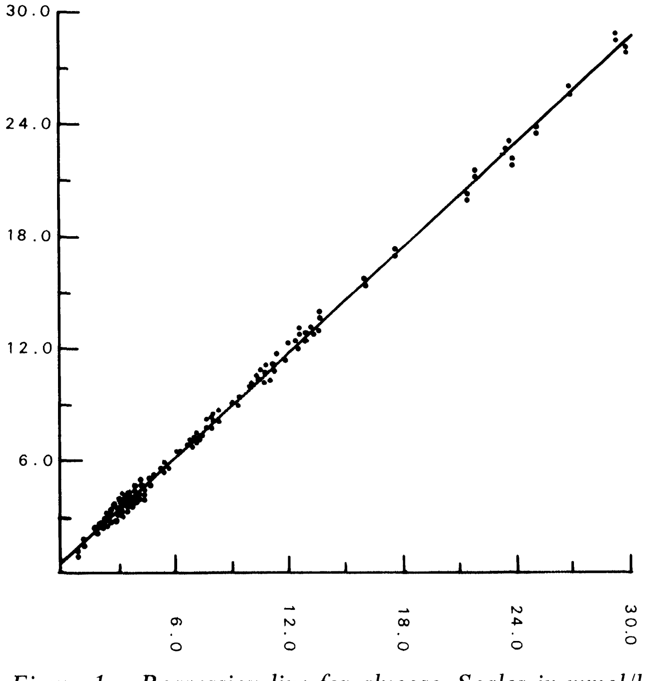

*Figure 1. Regression line for glucose. Scales in mmol/l. x = SMAC values (from comparison method), y = Hitachi values.*

*Table 10. Regression study. (Where x = routine method; y = studied method.)*

| | Pairs compared *N* | Range of values compared | Slope | Standard deviation of slope | *y*-intercept | Standard deviation of *y*-intercept | Residual variance | Coefficient of determination |
|---|---|---|---|---|---|---|---|---|
| Glucose | 110 | 2·6–28·8 | 0·93 | 0·01 | 0·34 | 0·06 | 0·0683 | 0·9916 |
| Urea | 110 | 2·8–29·8 | 0·93 | 0·01 | 0·59 | 0·06 | 0·1094 | 0·9966 |
| Creatinine | 59 | 36–98 | 0·89 | 0·04 | 3·51 | 3·25 | 29·0534 | 0·8768 |
| | 51 | 102–565 | 0·83 | 0·01 | 12·35 | 3·09 | 86·1424 | 0·9913 |
| Calcium | 110 | 1·6–2·6 | 1·00 | 0·02 | −0·04 | 0·04 | 0·0018 | 0·9621 |
| Protein | 110 | 41–80 | 1·04 | 0·02 | −1·64 | 1·43 | 2·7922 | 0·9538 |
| Amylase | 50 | 30–594 | 0·98 | 0·01 | 0·96 | 3·75 | 250·8259 | 0·9910 |

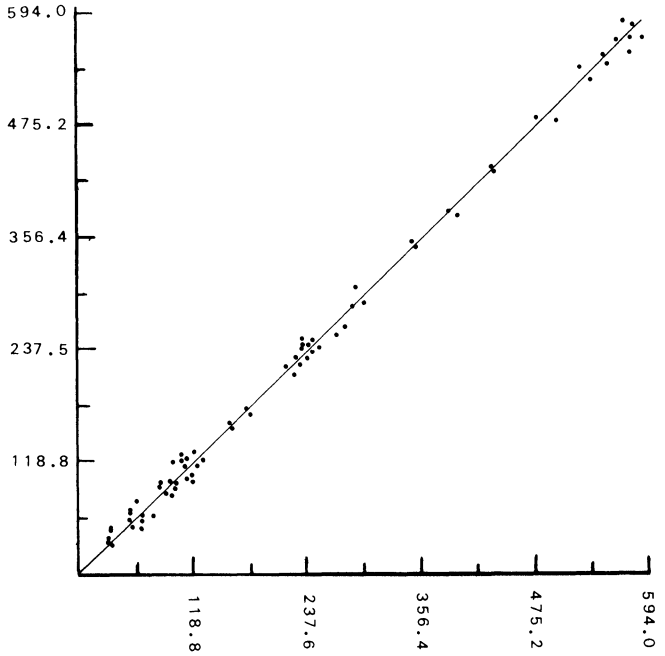

*Figure 2. Regression line for alpha-amylase. Scales in U/l. x = ABA-100 values (from comparison method), y = Hitachi values.*

*Reaction cuvette carry-over*

The coefficients of variation obtained in the reaction cuvette carry-over study were similar to those obtained when imprecision was investigated (table 7). The alpha-amylase example (table 9) shows the procedure followed for each analyte. Again, no appreciable carry-over was detected.

### *Relative accuracy*

Relative accuracy was investigated by the linear regression method [15] (table 10). Graphs showing the regression lines between the Hitachi results and those obtained from the Technicon SMAC and ABA-100 are shown for glucose (figure 1) and for alpha-amylase (figure 2). Similar graphs were obtained for the other analytes. Statistically, the regression lines show neither a proportional nor a systematic error for calcium, protein, and alpha-amylase, however, for glucose, urea, and creatinine a proportional and a systematic error exist (table 10).

### Conclusions

The Hitachi 705 analyser has proved to be a highly reliable and precise instrument for measuring the analytes studied. The coefficients of variation obtained are below those of clinical significance, according to Barnett [16], and within the range recommended by the SEQC.

The linearity of the methods is sufficiently broad to allow measurements of pathological levels without the necessity of diluting the sample except in extreme cases. Carry-over is insignificant. However, the comparative study of the Hitachi methodology with the SMAC and ABA-100 systems revealed only a good relative accuracy for calcium and protein (SMAC) and alpha-amylase (ABA-100). Calibration and programming are not overcomplicated, although training and experience is necessary, as with most automatic analytical systems. Only the very simple maintenance procedures, on a daily, weekly and monthly basis, are required.

The instrument can comfortably analyse 200–300 specimens/day, as required in a medium-size laboratory. Emergency samples may be given priority at any time.

The instrument, rather surprisingly, lacks a built-in sample-identification system: this facility might be expected considering the sophistication of the machine in other areas, i.e. the ease with which analytical methods may be modified, its versatility in accommodating kinetic tests, the elimination of priming before starting analyses, the small reaction and sample volumes needed, and the lack of requirement for commercial reagents.

### References

1. *Boletin Informativo of the Sociedad Española de Quimica Clinica*, **5** (1980).
2. Rauscher, E., v. Buelow, S., Neumann, U. and Schaich, E., paper presented at the 3rd International Congress on Clinical Enzymology (Salzburg, Austria, 1981).
3. Recommendations of the Deutsche Gessellschaft für Klinische Chemie, *Journal of Clinical Chemistry and Clinical Biochemistry*, **15** (1977), 249.
4. Szasz, G., Gruber, W. and Bernt, E., *Clinical Chemistry*, **22** (1976), 650.
5. Gruber, W., *Clinical Chemistry*, **24** (1978), 177.
6. Recommendations of the Deutsche Gessellschaft für Klinische Chemie, *Zeitung für klinische Chemie und klinische Biochemie*, **10** (1972), 182.
7. Würzburg, V., Hennrich, N., Lang, H., Prellwitz, W., Neumeier, D. and Knedel, M., *Klinische Wiesenscharft*, **54** (1976), 357.
8. Bartels, H. and Bohmer, M., *Clinica Chimica Acta*, **32** (1971), 81.
9. Trinder, P., *Annals of Clinical Biochemistry*, **6** (1969), 24.
10. Gutman, I. and Bergmeyer, H. U., in *Methods of Enzymatic Analysis*, Ed. Bergmeyer, H. U. (Verlag Chemie Weinheim and Academic Press, Inc., New York and London, 1974), 1791.
11. Ray Sarkar, B. C. and Chanhan, U. P. S., *Analytical Biochemistry*, **20** (1967), 155.
12. Weichselbaum, T. E., *American Journal of Clinical Pathology*, **16** (1946), 40.
13. Knedel, M., personal communication.
14. *Product Labelling of SMAC* (Technicon Instruments Corporation, Tarrytown, New York, 1974).
15. Westgard, J. O. and Hunt, M. R., *Clinical Chemistry*, **19** (1973), 49.
16. Barnett, R. N., *American Journal of Clinical Pathology*, **50** (1968), 671.

